# Postbiotics: Multifunctional Microbial Products Transforming Animal Health and Performance

**DOI:** 10.3390/vetsci12121191

**Published:** 2025-12-12

**Authors:** Sahdeo Prasad, Bhaumik Patel, Prafulla Kumar, Rajiv Lall

**Affiliations:** 1 RD Life Sciences LLC, 13707 66th Street N, Largo, FL 33771, USA; 2 Department of Immunotherapeutics and Biotechnology, Texas Tech University Health Science Center, Abilene, TX 79601, USA; bhaumik.patel@ttuhsc.edu

**Keywords:** postbiotics, antimicrobial, antioxidant, immunomodulatory, livestock animals

## Abstract

Postbiotics, which are preparations of inanimate microorganisms and their components, have emerged as a promising functional ingredient in animal health and nutrition. Postbiotics are primarily composed of microbial cell fractions, metabolites, enzymes, vitamins, polysaccharides, and short-chain fatty acids. Unlike probiotics, postbiotics do not contain live microorganisms, which strengthens their greater stability and safety in feed/food formulations. Postbiotics offer several beneficial effects, including antioxidant, anti-inflammatory, immune-modulatory, and antimicrobial actions. They enhance antioxidant enzymes, neutralize reactive oxygen species, and inhibit lipid peroxidation, thereby protecting tissues from oxidative damage. Postbiotics also inhibit pro-inflammatory molecules like TNF-α and IL-6, while enhancing the anti-inflammatory cytokine IL-10, promoting the maturation and function of immune cells, and increasing secretory IgA production. They suppress a variety of pathogenic bacteria, including *Escherichia coli*, *Salmonella*, *Staphylococcus*, *Campylobacter*, etc., both in vitro and in vivo. Moreover, they increase beneficial gut bacteria and improve the digestion and integrity of the intestine. This article outlines the beneficial effects of postbiotics in animals including poultry, swine, canine, feline, horses, and ruminant animals, either as feed/food or as a supplement. The integration of postbiotics into animal feed improves growth performance, feed conversion ratios, and disease resistance in animals. Thus, the multifunctional benefits of postbiotics make them a valuable tool for healthy companion animals and sustainable livestock production, supporting both animal welfare and productivity without the drawbacks associated with antibiotic growth promoters.

## Simple Summary

Postbiotics are non-living cell composites that possess functional and beneficial properties to animal and human health and nutrients. Postbiotics not only improve gut biota, digestion, and intestinal health but also suppress various pathogenic bacteria. These benefits are not limited to the digestive system; it also acts as an antioxidant, reduces inflammation, and strengthens immunity. Particularly, it plays a significant role in animal farming, especially for ruminants, poultry, and swine, as well as the care of companion animals such as canines, felines, and horses. Postbiotics are used as a feed/food additive or as a supplement to enhance health, welfare, productivity, and resistance to antibiotics in livestock and companion animals. Thus, postbiotics are a multifunctional byproduct that helps in transforming animal health.

## 1. Introduction

Livestock have a great importance in human life and in the food chain, as they are associated with feeding the world’s rapidly expanding population. Livestock are a source of essential food products such as milk and meat, as well as non-food goods like hides and fertilizers, representing almost half the value of agricultural production worldwide. The value of livestock products is predicted to continue increasing because of their sustainable rise in demand [1]. It is estimated that the global consumption of meat may increase 73% and dairy products approximately 58% by 2050 [2]. Because of this increased demand, UN-FAO and the World Bank expect a large and rapid increase in animal populations. They predict an increase in the global cattle population from 1.5 billion to 2.6 billion animals, and the global goat and sheep population from 1.7 billion to 2.7 billion animals by the year 2050 [3].

However, increasing the population of livestock may cause various issues that include an elevated pressure on natural resources and climate changes like elevated temperatures, droughts, and deforestation. Therefore, to maintain the supply and demand of animal products, an increase in livestock productivity is required rather than an increase in animal numbers. Increasing livestock productivity faces many challenges, which include, but are not limited to, adequate feed supplies, appropriate superintendence, protecting livestock from disease, veterinary research, and balancing economic, environmental, and social concerns [4]. Among various factors, livestock diseases remarkably affect the productivity of livestock, causing a loss in production systems through morbidity, mortality, and prevention and control costs. Based on the World Organization of Animal Health’s (2023) estimate, animal diseases contribute to around 20% of the annual loss in livestock productivity, impacting meat and dairy production.

Livestock encounter a variety of diseases throughout their lives, primarily caused by pathogenic viral and bacterial infections. In addition, livestock suffer from other health problems such as arthritis, ketosis, mastitis, fertility, gut leakage, and gut microbiota imbalance [5,6]. Various preventive and therapeutic practices can be implemented to control the incidence of diseases, as well as livestock growth and productivity, including the use of antibiotics. However, the continuous overuse of antibiotics may result in antimicrobial drug resistance, which can contribute to adverse global impacts [7]. Considering this global public health threat, many countries have restricted the use of antibiotics as growth promoters and have encouraged the development of alternatives to antibiotics in veterinary medicine and animal farming. Various natural products, such as phytochemicals, probiotics, postbiotics, enzymes, antimicrobial peptides, bacteriophages, clay, and metals, have been promoted as an alternative to antibiotics in animal production [8]. These natural products also promote growth, stimulate the immune system, relieve oxidative stress, and improve gut health and other body systems to promote health.

Companion animals also suffer from various health issues, such as arthritis, cancer, diabetes, and dental problems, as well as infection, obesity, allergies, and trauma. Since approximately 70% of U.S. households own at least one pet, their health and wellbeing are very important [9]. A proper diet plays a crucial role in the health of companion animals, as unhealthy nutrition is often linked to a poor immune system, aging, and improper digestion. Considering these facts, various types of pet foods include phytochemicals, supplements, and minerals, which are being used in their food to increase immunity, promote digestion, reduce inflammation, and combat oxidative stress. In this review, the beneficial effects of postbiotics in companion animals and livestock health and productivity are described.

## 2. Prebiotics, Probiotics, and Postbiotics

Prebiotics, probiotics, and postbiotics are natural substances used to provide health benefits to humans, companion animals, and livestock. Prebiotics are plant-derived non-digestible carbohydrates that exhibit a selective activity towards the stimulation of metabolism and proliferation of intestinal beneficial bacteria, thereby improving the composition of the gut microbiota [10]. Prebiotics not only provide nutrients and promote the growth of beneficial gut microbiota but also decrease the relative abundance of pathogenic bacteria [11].

Probiotics are beneficial live microorganisms that confer a health benefit on the host when administered in adequate amounts. Although probiotics are claimed to provide health benefits by improving or restoring gut microbiota, they may also cause adverse effects. Since probiotics are live microorganisms, they may interact with hosts [12,13]. It has been reported that probiotics have the possibility to cause opportunistic systemic and local infections, detrimental immunological effects, metabolic disturbances, and allergic reactions and facilitate the spread of antimicrobial resistance [14]. Moreover, probiotics can cause adverse effects to consumers with immunodeficiency, short bowel syndrome, central venous catheters, and cardiac valve disease. For example, risks can occur due to the passage of viable bacteria from the gastrointestinal tract to the internal organs in consumers with inflammatory bowel disease [15].

Considering the limitations and potential side effects of probiotics, postbiotics are identified as a safer biologically active agent. Postbiotics are an emerging product for promoting companion animal and livestock health and are also fairly advanced compared to prebiotics and probiotics. The International Scientific Association of Probiotics and Prebiotics (ISAPP) in the year 2021 proposed a definition of postbiotics as a “preparation of inanimate microorganisms and/or their components that confers a health benefit on the host” [16]. Although postbiotic is the latest terminology, it was previously termed as paraprobiotics, parapsychobiotics, ghost probiotics, metabiotics, tyndallized probiotics, and bacterial lysates. 

## 3. Components of Postbiotics

**Figure 1:**
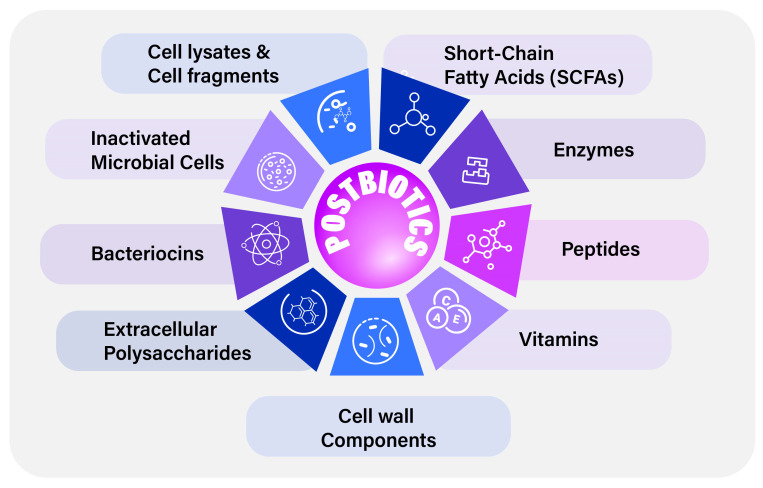
Components of postbiotics.

Postbiotics constitute multiple components (Figure 1), from large cellular fractions to small molecules of metabolites. These components may work directly or indirectly, but provide beneficial effects.

***Short-chain fatty acids (SCFAs)***

SCFAs are fatty acids of two to six carbon atoms. They are produced by gut bacteria during the fermentation of dietary fiber and indigestible starch in the large intestine [17]. Although the most common SCFAs are acetate, propionate, and butyrate, other SCFAs are isobutyric acid, valeric acid, isovaleric acid, and 2-methylbutyric acid. SCFAs are considered to be the main energy source of colonocytes (the epithelial cells of the colon), making them crucial to gastrointestinal health. Among other SCFAs, butyrate has a significant value for the colon, as it provides energy to colonocytes [18]. Thus, SCFAs play diverse roles in maintaining physiological functions such as gut health and immune function [19].

***Enzymes***

Microorganisms like bacteria or yeast produce enzymes through various fermentation processes, including submerged and solid-state fermentation. These enzymes have the ability to break down complex carbohydrates and other molecules, aiding in digestion and nutrient absorption. The key enzymes include amylases, glucoamylases, and pullulanase, which work together to convert starch into fermentable sugars [20]. These enzymes have diverse roles in animal feed digestion and gut health that facilitate industrial feed development and enhance animal health and wellness.

***Peptides***

Peptides are short chains of amino acids and have different biological functions. They originate from both exogenous and endogenous sources and are obtained through microbial fermentation processes [21]. Endogenous peptides are derived from microbial cells, while exogenous peptides are derived before fermentation begins. They possess functions that range from antimicrobial, immunomodulatory, and antioxidant properties. Bioactive peptides contribute significantly to gut health by influencing barrier integrity and gut microbiota composition, regulating immune responses, and showing enzymatic activities that impact digestion [22].

***Vitamins***

Some probiotic bacteria and yeast can produce essential vitamins, including vitamin B (B1, B2, B6, B12) and Vitamin K. Vitamins are key nutrients that help maintain the normal health status of animals and can be included in postbiotics through the fermentation of microbes.

***Cell wall components***

Cell wall components derived from probiotic bacteria constitute significant postbiotic elements with diverse biological activities. These primarily include teichoic acids, lipoteichoic acids, and peptidoglycan-derived muropeptides [22]. Research demonstrates that cell wall components from beneficial bacteria can suppress pathogen-induced inflammatory responses while promoting regulatory immune mechanisms [23].

***Extracellular polysaccharides***

Extracellular polysaccharides (EPSs) are complex carbohydrates secreted by probiotic bacteria that form an important class of postbiotics. These high-molecular-weight polymers are strain-specific and display a great structural diversity, ranging from homopolysaccharides to heteropolysaccharides with varying compositions and linkages [24]. Research indicates that EPSs from lactic acid bacteria possess antimicrobial, antitumor, anti-biofilm, antiviral, anti-inflammatory, and immunomodulatory properties [25]. Thus, EPSs can influence gut microbiota composition, increase the growth of beneficial bacteria, and inhibit pathogens.

***Bacteriocins***

Bacteriocins are found to be antimicrobial peptides produced by various bacteria that constitute an important class of postbiotics with significant therapeutic potential. These ribosomally synthesized peptides exhibit antimicrobial activity with a variable spectrum depending on the peptide structure, targeting several pathogenic bacteria while sparing beneficial microorganisms [26]. Bacteriocins function through multiple mechanisms, including pore formation in cell membranes, the inhibition of cell wall synthesis, and the disruption of cellular metabolism. Research demonstrates that bacteriocins contribute to pathogen suppression in the gut microbiome and may enhance innate immunity [27]. Their stability, specificity, and potency make bacteriocins promising alternatives to conventional antibiotics, particularly in addressing antibiotic resistance.

***Inactivated microbial cells***

Inactivated microbial cells are non-viable probiotic bacteria that retain their beneficial properties and serve as important postbiotic components. Despite being non-viable, inactivated microbial cells maintain their immunomodulatory and anti-inflammatory properties through interactions with host immune receptors. Research demonstrates that heat-killed probiotics can enhance intestinal barrier function, modulate cytokine production, and regulate immune responses [28]. The advantages of using inactivated cells include enhanced stability, extended shelf life, and reduced safety concerns compared to live probiotics.

***Cell fragments***

Cell fragments are structural components of bacterial cells that remain after cell lysis or disruption and represent valuable postbiotic elements with distinct biological activities. These fragments primarily consist of cell wall components, membrane structures, and intracellular organelles that retain their immunomodulatory and anti-inflammatory properties even after the cell is no longer viable. Studies have demonstrated that fragments from beneficial bacteria can suppress pathogen-induced inflammation while promoting regulatory immune mechanisms [29]. The composition and bioactivity of cell fragments vary depending on the bacterial strain, growth conditions, and fragmentation method.

***Cell lysates***

Cell lysates are preparations of bacterial cells that have been broken open to release their intracellular contents, creating a complex mixture of bioactive molecules with diverse functional properties. These lysates contain enzymes, peptides, nucleic acids, vitamins, and other cellular components that collectively contribute to their biological activity. Research demonstrates that bacterial lysates exhibit immunomodulatory, anti-inflammatory, and antimicrobial properties, making them valuable in both therapeutic and nutritional applications [30].

## 4. Physiological Effects of Postbiotics

**Figure 2:**
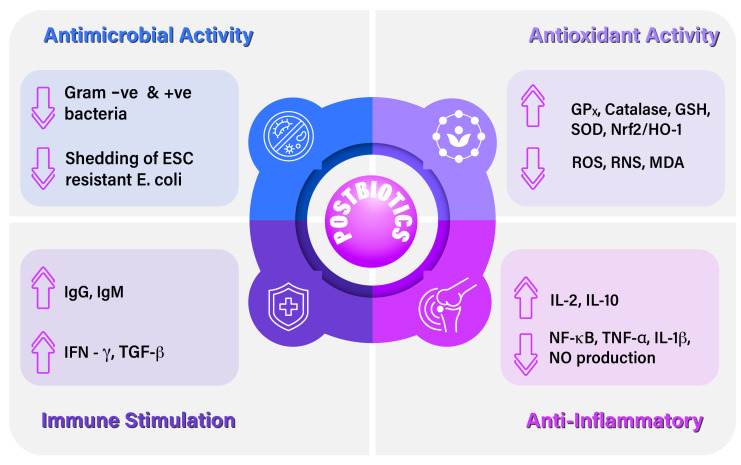
Physiological effect of postbiotics. ↑ arrow indicates increase and ↓ arrow indicates decrease.

The application of postbiotics has emerged in various foods and supplements due to their vast physiological properties, including antioxidant, anti-inflammatory, immune stimulatory, antimicrobial, and gut health support activity (Figure 2).

### 4.1. Antioxidant Activity

Postbiotics are an emerging and promising source of antioxidants and have shown potential health benefits. The antioxidative properties of postbiotics have been extensively investigated both in murine models and in vitro. They upregulate host antioxidant activity, modulate signaling pathways, and reduce reactive oxygen species (ROS) levels. In a study, dietary feed postbiotics from *Lactobacillus plantarum* RG14, RG11 and TL1, in post-weaning lambs demonstrated an antioxidant activity by increasing glutathione peroxidase (GPX) in serum and ruminal fluid and decreasing serum thiobarbituric acid reactive substances (TBARS). The *L. plantarum* postbiotic also upregulated hepatic GPX1, GPX4, copper, and zinc superoxide dismutase (SOD) in the lambs [31]. In another study, the postbiotic from *L. casei* exhibited the most potent antioxidant activity, followed by *L. rhamnosus*, *L. fermentum*, *L. acidophilus*, and *L. plantarum* [32]. In Wistar rats, postbiotics from *L. acidophilus* and *L. plantarum also* showed antioxidant activity. Moreover, animals fed with postbiotic-fortified yogurt exhibited significant antioxidant effects. The antioxidant capacity of yogurt increasingly peaked at 48.81% on day 14 [33].

Postbiotics from *L. plantarum* O7S1 have shown a remarkable antioxidant and scavenging ability against DPPH (2,2-Diphenyl-1-picrylhydrazyl), hydroxyl, and superoxide radicals, with inhibition rates of 88.78, 78.91, and 34.85%, respectively. However, it was found that cell-free supernatant postbiotics have a comparatively higher antioxidant capacity than exopolysaccharides (EPSs) [34]. Additionally, postbiotics derived from *L. rhamnosus*, *L. reuteri*, and their combination have shown significant antioxidant properties, as assessed by using the DPPH assay [35]. In heat-stressed broilers, the supplementation of a basal diet with 0.3% of postbiotics from *L. plantarum* strains, RI11, RS5, or UL4 relieved broilers from oxidative stress. Postbiotics supplementation, especially with the RI11 strain, increased the total antioxidant capacity (TAC), as well as catalase and glutathione (GSH) antioxidant enzymes in heat-stressed broilers. In addition, meat malondialdehyde (MDA) was found to be lower in the postbiotic groups [36]. This study indicates that natural sources of antioxidants from postbiotics are beneficial for heat-stressed broilers.

The postbiotics from *Bacillus amyloliquefaciens* and *Lactiplantibacillus plantarum* have shown antioxidant activity by scavenging and reducing the activity of hydroxyl radicals, DPPH, and ABTS radicals [37]. *Lacticaseibacillus casei* CRL431 postbiotics were also able to exert antioxidant activity in Aflatoxin B_1_-induced oxidative stress in rat models. CRL431 postbiotics exhibited a reduced oxidative stress by increasing GPX and catalase activities, as well as reducing the production of H_2_O_2_. It has been suggested that *Lacticaseibacillus casei* postbiotics have the ability to permeate intestinal barriers to enter the bloodstream and combat AFB_1_-induced oxidative stress [38]. Postbiotics (yeast cell wall, hydrolyzed yeast) with other components like clay and phytogenic feed additives have been shown to decrease the biomarkers of oxidative stress in sows. In mycotoxin-challenged pregnant and lactating sows, the administration of these agents in feed modulated TBARS, protein carbonyls, and TAC, indicating its beneficial effects on oxidative stress biomarkers [39].

The *L. kunkeei* culture supernatant exhibited antioxidant effects in an experimental cell culture model. The pretreatment of this supernatant increased the expression of Nrf2/HO-1 (nuclear factor erythroid 2-related factor 2/heme oxygenase-1), a key player in cellular defenses against oxidative stress. This antioxidant activity was aligned with its robust ability to remove H_2_O_2_-induced ROS [40]. *Saccharomyces cerevisiae* fermentation product (SCFP) postbiotics showed beneficial effects in weanling pigs. This postbiotic decreased MDA and increased the catalase, total SOD, and TAC, indicating an enhanced antioxidative capacity [41]. The *L. paracasei* postbiotic decreased the oxidative stress in a rat model where a high-fat diet-induced decrease in the activities of the antioxidant enzymes (GPX, glutathione-s-transferase, glutathione reductase, SOD) was recovered through postbiotic administration. In addition, the increased level of MDA in the highly obese group was found to be restored by postbiotics [42]. The postbiotics from *Cetobacterium somerae* and *Lactococcus lactis* also induced antioxidant levels in fish. The feeding of these postbiotic diets has been shown to improve the health of fish remarkably, probably by improving mucosal C3, TAC, and SOD activities and decreasing lipid peroxidation product MDA levels [43].

In addition, EPSs from *L. plantarum* R301 exhibited strong antioxidant activity. The scavenging rate of EPSs was found to be up to 100% at concentrations of 4 mg/mL. Moreover, EPSs also exerted a radical reducing power, which was about 30% that of ascorbic acid [44]. In an ibuprofen-exposed mice model, postbiotic EPSs were found to enhance the activity of liver antioxidant enzymes (SOD and catalase), increase GSH content, and reduce NO, MPO, and MDA levels, demonstrating a protective effect against ibuprofen-induced oxidative stress and inflammatory response [45]. Another postbiotic component, sodium butyrate, has also shown antioxidative properties. A sodium butyrate-containing diet, when fed to adult cats, has been shown to increase GPX levels and decrease MDA content compared with a control diet [46]. Table 1 summarizes the antioxidant properties of postbiotics in brief.

**Table 1:** Antioxidant activity of postbiotics.

Antioxidant Effects	Model	Dose	Reference
Postbiotics of *L. plantarum* RG14 increase GPX in serum, upregulate hepatic GPX1, GPX4, and SOD	Post-weaning lambs	0.9% (*v*/*w*)	[31]
*Lactobacillus casei* exhibits potent antioxidant activity	DPPH assay	0.5 mL	[32]
*L*. *acidophilus* BLAC 258 and *L. plantarum* from yogurt increased antioxidant activity up to 48.81%	DPPH assay	ND	[33]
Postbiotics from *Lactiplantibacillus plantarum* O7S1 scavenge ROS and show antioxidant activity	DPPH and radical scavenging assays	0.5 and 1.0 mL	[34]
Postbiotics from different lactobacillus induce antioxidant activity	DPPH assay	ND	[35]
*Lacticaseibacillus casei* CRL431 postbiotics reduce H_2_O_2_ levels, oxidative stress, and increase GPx and catalase activities	Rat	ND	[38]
Postbiotics from *L. kunkeei* upregulate antioxidant markers, Nrf2/HO-1, and quench ROS	HaCaT cells	2.5%, 5%, and 10%	[40]
*Lactobacillus paracasei* postbiotic restored antioxidant enzymes GPX, GST, GR, and SOD and decreased MDA	Albino Wistar rat	100 and 200 mg/kg	[42]
Postbiotics from *Cetobacterium somerae* and *Lactococcus lactis* improve T-AOC and SOD activities and decrease MDA level	Common carp (Cyprinus carpio) fish	0.2 and 0.3 g/kg	[43]
EPS postbiotics enhance SOD, catalase, and GSH and reduced NO, MPO, and MDA levels	Rats	200 mg/kg	[45]

AOC—antioxidant capacity; EPS—exopolysaccharide; GPX—glutathione peroxidase; GSH—glutathione; SOD—superoxide dismutase; MDA—malondialdehyde; MPO—myeloperoxidase; ND—mot determined; NO—nitric oxide; Nrf2/HO-1—nuclear factor erythroid 2-related factor 2/heme oxygenase-1; ROS—reactive oxygen species.

### 4.2. Anti-Inflammatory

Inflammation is associated with several chronic diseases and impacts the overall health of livestock. Postbiotics have shown anti-inflammatory effects in livestock and several other experimental models (Table 2). In a study, heat-killed *Lactiplantibacillus argentoratensis* BBLB001 exhibited anti-inflammatory effects in a Caco2/RAW264.7 cell co-culture and in dextran sodium sulfate (DSS)-induced colitis mouse models. These postbiotics markedly reduced IL-8 secretion in a Caco-2 cell culture medium after the lipopolysaccharide (LPS)-mediated stimulation of RAW264.7 cells [47]. Additionally, postbiotics from the *B. amyloliquefaciens* J and *Lactiplantibacillus plantarum* SN4 combination have shown a reduction in NO secretion in RAW 264.7 macrophage cells in response to LPS-induced inflammation [37]. Postbiotics from heat-killed *Bifidobacterium bifidum* B1628 exerted an anti-inflammatory activity in DSS-induced colitis in mice. In brief, postbiotic supplementation decreased the serum levels of pro-inflammatory cytokines (IL-1β and TNF-α) and increased the levels of an anti-inflammatory cytokines (IL-13) compared with the DSS group, suggesting its anti-inflammatory activity in animals [48]. 

*S. boulardii* has been widely used in food and pharmaceutical research due to its anti-inflammatory properties and gut health benefits. It has been observed that the oral administration of heat-killed *S. boulardii* postbiotics and *S. boulardii* β-glucan decreased the levels of pro-inflammatory cytokines (TNF-α, IL-1β, and IL-6) in the serum, and suppressed the expressions of TNF-α, IL-1β, and IL-6 mRNA in the colon of DSS-exposed mice, exhibiting the suppression of inflammation [49]. The findings from in vitro assays showed that *L. plantarum* CRL 759 postbiotics significantly reduced the production of IL-6, TNF-α, and NO in RAW 264.7 cells stimulated with LPS, which indicates its anti-inflammatory actions [50]. In a high-fat diet-fed zebrafish model, the postbiotics of SWFC (cultured supernatant mixture of *Cetobacterium somerae* and *Lactococcus lactis*) showed anti-inflammatory effects by reducing the expression level of pro-inflammatory factors (NF-κB, TNF-α, and IL-1β) and increasing the expression level of anti-inflammatory factor IL-10 [51]. Heat-killed *Pediococcus pentosaceus* PP18 postbiotics were also found effective in mitigating intestinal injury and inflammation in zebrafish fed on a high-fat diet. This postbiotic supplementation markedly downregulated the pro-inflammatory cytokines TNF-α, IL-6, and IL-1β, reducing intestinal inflammation [52]. 

The postbiotic lactic acid bacteria lysates (LAB-P) prepared from *Lactiplantibacillus plantarum* K8 exhibited an anti-inflammatory response in obesity-induced high-fat diet (HFD)-fed mice. The LAB-P postbiotics inhibited pro-inflammatory cytokines (IL-1β and IL-6) and downregulated NF-κB expression, which helped in the prevention of obesity [53]. *Bifidobacterium bifidum* postbiotics also showed anti-inflammatory activity in chicken small intestinal epithelial cells. These postbiotics reduced the inflammatory cytokines, including IL-1β and IL-8, while increasing anti-inflammatory cytokines, such as IL-10 and IL-4, thereby mitigating inflammation [54]. Moreover, *L. plantarum* culture supernatants (postbiotics) exert an anti-inflammatory activity in dendritic cells by suppressing the release of IL-12p70 and increasing the release of the anti-inflammatory IL-10 cytokine in vitro. Xu et al. [55] also demonstrated that *S. boulardii* freeze-dried and spray-dried postbiotics displayed an anti-inflammatory activity in dextran sulfate sodium (DSS)-induced ulcerative colitis in mice. These postbiotics inhibited inflammatory factors IL-1β, IL-6, and TNF-α and increased anti-inflammatory cytokine IL-10 in the blood and colons of mice.

The postbiotic compound EPS from *L. plantarum* R301 has also demonstrated a decent anti-inflammatory activity by inhibiting LPS-induced inflammation in RAW 264.7 cells. It decreased NO production and the expression of the pro-inflammatory cytokine IL-6 [44]. The study showed that EPSs obtained from *L. plantarum* exert anti-inflammatory effects through the suppression of pro-inflammatory mediators, such as cyclooxygenase-2 (COX-2), IL-6, TNF-α, and IL-1β, and the downregulation of an inducible nitric oxide synthase, induced through LPS administration in RAW 264.7 cells [56]. Postbiotic Urolithin obtained from microbiota cultures was tested for its inflammatory response in THP-1-derived macrophages, RAW 264.7 macrophages, peripheral blood mononuclear cell (PBMC)-derived macrophages, and primary neutrophils. Urolithin A suppressed the LPS-induced inflammatory response by inhibiting TNF-α attenuation and inducing IL-10 [57]. A soluble protein, HM0539, derived from *L. rhamnosus* GG has been shown to play a role in modulating inflammatory responses. In LPS-stimulated RAW 264.7 macrophages and in DSS-induced murine colitis models, the HM0539 protein inhibited inflammatory molecules COX-2, inducible nitric oxide synthase (iNOS), prostaglandin E2 (PGE2), NO, and NF-κB [58].

**Table 2:** Anti-inflammatory effects of postbiotics.

Anti-Inflammatory Effects	Model	Dose	Reference
Heat-killed *L. argentoratensis* dried postbiotics reduce IL-8 secretion	Caco2/RAW264.7 cells and mice	500 μg/mL in vitro, 0.002–0.01% in mice	[47]
Inhibition of NO induced by LPS	RAW 264.7 macrophage cells	0.5–4 mg/mL	[37]
Heat-killed *S. boulardii* postbiotics suppress TNF-α, IL-1β, and IL-6	C57BL/6J mice	200 μL	[49]
Postbiotics *Cetobacterium somerae* and *Lactococcus lactis* reduce expression of NF-κB, TNF-α, and IL-1β	High-fat diet-fed zebrafish model	0.3 g/kg	[51]
Heat-killed *Pediococcus pentosaceus* PP18 postbiotics reduce intestinal inflammation by reducing TNF-α, IL-6, and IL-1β	High-fat diet zebrafish	10^7^ CFU/gm of heat-killed	[52]
Lysates of *Lactiplantibacillus plantarum* K8 postbiotics inhibit IL-1β, IL-6, and NF-κB expression	Obesity-induced high-fat diet-fed mice	100 mg/kg	[53]
Postbiotics from *S. boulardii* reduce pro-inflammatory factors and increase anti-inflammatory factors	DSS-induced C67BL/6J colitis mouse	ND	[55]
EPSs from *Lactobacillus plantarum* R301 decrease NO production and pro-inflammatory cytokine Il-6	RAW 264.7 cells	20–1000 μg/mL	[44]
EPSs from *L. plantarum* block inflammation and TLR4	LPS-induced RAW 264.7 cells	50, 100, 200 μg/mL	[56]
HM0539 from *Lactobacillus rhamnosus*	LPS-stimulated RAW 264.7	25, 50, 100 ng/mL	[58]

EPS—exopolysaccharide; IL—interleukin; NF-κB—nuclear factor kappa B; NO—nitric oxide; ND—not determined; TLR4—toll-like receptor 4; TNF—tumor necrosis actor.

### 4.3. Antimicrobial

Postbiotics show remarkable antimicrobial properties (Table 3). In one study, postbiotic from *Lactiplantibacillus plantarum* RG14, RI11, and UL4 exhibited a remarkable inhibitory activity against *Pediococcus acidilactici* 446, *Escherichia coli* E-30, *Salmonella enterica* CS3, and vancomycin-resistant *Enterococci* [59]. Later, Aliouche showed that cell-free supernatant postbiotics from *Lactiplantibacillus plantarum* O7S1 have a significant antimicrobial activity against both Gram-positive and Gram-negative pathogens [34]. Additionally, the *L. plantarum* PA21 cell-free supernatant postbiotics exhibited antimicrobial activity against all nine methicillin-resistant *Staphylococcus* aureus (MRSA) and three out of thirteen Klebsiella pneumoniae clinical isolates [60]. Thus, *L. plantarum* PA21 postbiotic metabolites proved to be a prolific source of antimicrobial agents against multidrug-resistant pathogens.

Postbiotics from *Pediococcus acidilactici*, *Latilactobacillus sakei*, and *Staphylococcus xylosus* have also been shown to exert antimicrobial activity. The solution of 5% and 10% of these postbiotics with EDTA decreased the number of *L. monocytogenes* in growth media. In addition, *Pediococcus acidilactici* postbiotics (10%) decreased the *S. typhimurium* and *L. monocytogenes* counts in chicken drumsticks [61]. Thus, postbiotics may be an alternative approach to reducing food-borne pathogens and extending the shelf life of poultry meat and meat products. Postbiotics from *L. rhamnosus* and *L. reuteri* have exhibited antimicrobial properties against *E. coli* and *S. aureus* pathogens. It has been observed that the addition of postbiotics (*L. rhamnosus* and *L. reuteri*) to meat samples displayed a reduction in *E. coli* and *S. aureus* [35]. Similarly, postbiotic-incorporated films also showed an antimicrobial activity against *L. monocytogenes*. The postbiotic-incorporated films reduced the counts of *L. monocytogenes* in ground meat. In addition, meat wrapped in postbiotic film displayed a significant decrease in the total mesophilic and psychrophile counts [62].

The postbiotics from *L. acidophilus* and *S. cerevisiae* have shown an impact on the shedding of extended-spectrum cephalosporin (ESC)-resistant *E. coli* in pigs. Feeding with these postbiotics demonstrated a reduction in the shedding of ESC-resistant *E. coli* in pigs, indicating positive impacts on the suppression of pathogenic bacteria [63].The postbiotic components, enterocins, also displayed an antibacterial and antibiofilm effect on pathogenic methicillin-resistant *Staphylococcus* aureus (MRSA) strains, exhibiting a high inhibition on biofilm formation, indicating that enterocins offer a promising option for the prevention and treatment of bacterial infections caused by biofilm-forming MRSA [64]. Furthermore, in Wistar rats fed with postbiotic-fortified yogurt, a decrease in the *L. monocytogenes* count by approximately 2 Log_10_ was observed on day 3 due to the postbiotic yogurt [33]. In another study, *L. casei* postbiotics exhibited the suppression of various pathogenic bacteria like *Salmonella enterica*, *L. monocytogenes*, and *Staphylococcus aureus*, as observed through agar disk diffusion assays [32].

**Table 3:** Antimicrobial effects of postbiotics.

Antimicrobial Effect	Model	Dose	Reference
Postbiotics from *Lactiplantibacillus plantarum* O7S1 inhibit Gram-positive and Gram-negative pathogens	Agar well diffusion assay	1 mg/mL	[34]
*Lactobacilli*-derived postbiotics inhibit *E. coli* by 5.54 log Colony forming unit (CFU)/g	Meat samples	100 mL/mg	[35]
Postbiotic-incorporated bacterial nanocellulose films reduce ~5 log cycles of *L. monocytogenes* count	Ground meat	21.21%	[62]
Postbiotic-fortified yogurt decreases *L. monocytogenes* count by ~2 Log_10_	Wistar rats	1.0 g	[33]

### 4.4. Immune Stimulation

Postbiotics play a crucial role in modulating the immune response by stimulating immune cells and factors associated with immunity within the body. The stimulation of immune responses by postbiotics has been observed in both in vitro and murine models (Table 4). In a study, the supplementation of 0.3% postbiotics harvested from *L. plantarum* increased the serum IgA and IgG content in growing mink, suggesting that dietary postbiotics have the potential to improve growth performance and immune status [65]. Also, the oral administration of extracellular vesicle (EV) postbiotics isolated from *L. rhamnosus* GG (LGG-EV) improved anti-PD-1 immunotherapy efficacy against colorectal cancer. LGG-EV modulated intestinal immunity by increasing the CD8^+^ T/CD4^+^ T cell ratio in mesenteric lymph nodes and enhancing the ratio of MHC II^+^ DC cells, CD4^+^ T cells, and CD8^+^ T cells in tumor tissues [66]. It was also observed that the daily administration of EVs from probiotic and commensal *E. coli* strains to healthy suckling rats increases humoral and cellular immunity. The postbiotic EVs exhibited higher levels of plasma IgG, IgA, and IgM and a greater proportion of T cells, NK, and NKT cells in the spleen of rats. Thus, postbiotic EVs have a role in increasing immune function [67].

Natural killer (NK) cells play a key role in innate and adaptive immunity. Postbiotic treatment caused NK cell activation in stress-induced mice in accordance with Th1/Th2 expression levels [68]. Postbiotics from *Bacillus velezensis* Kh2-2 lysates demonstrated the ability to stimulate immune activation in macrophages. This postbiotic upregulated NF-κB and MAPK signaling pathways and promoted immune-related cytokine secretion. In addition, this lysate stimulated proliferation and polarized Th1 response by inducing the production of IL-2 and IFN-γ and inhibiting IL-10 expression in splenocytes [69]. 

Postbiotics improve the growth performance of broilers under heat stress by increasing their immune status. Feeding a 0.3% mixture of postbiotics to broilers (produced from different *L. plantarum* strains, and defined as RI11, RS5, and UL4) increased plasma IgM, IgG, and IgA levels [70]. Postbiotics also affect the expression of the immune-related genes of white shrimp. The supplementation of heat-killed postbiotic *Pediococcus pentosaceus* PP4012 has been found to increase the expression of immune-related genes alf, pen3a, and pen4 in shrimp, which leads to an increase in the shrimp’s resistance to pathogens [71].

**Table 4:** Immune stimulation effects of postbiotics.

Immune Stimulation	Model	Dose	Reference
*L. plantarum* postbiotics increase IgA and IgM content and decrease TNF-α and IL-8 levels	12-week-old mink	0, 0.15%, 0.3%, and 0.45%	[65]
Extracellular vesicle postbiotics from *Lactobacillus rhamnosus* increase CD8^+^ T/CD4^+^ T cell ratio and MHC II^+^ DC cells	Mice	ND	[66]
Postbiotic EVs increase plasma IgG, IgA, and IgM and proportion of Tc, NK, and NKT cells in the spleen	Suckling rats	N/A	[67]
Postbiotics mixture of *L. plantarum* KM1, *L. Plantarum* KM2, and *B. velezensis* KMU01 activates NK cells in accordance with Th1/Th2 expression level	C57BL/6N mice	400 μL	[68]
Postbiotics from *Bacillus velezensis* Kh2-2 lysates downregulate NF-κB and MAPK signaling, IL-2, and IFN-γ and increase IL-10 expression	RAW264.7 cell lines and mice.	25–100 μg/mL, 10^6^ CFU/Kg/day	[69]

IFN—interferon; Ig—immunoglobulin; IL—interleukin; LPS—lipopolysaccharide; MAPK—mitogen-activated protein kinase; N/A—not applicable; ND—not determined; NF-κB—nuclear factor kappa B; NK—natural killer; TNF—tumor necrosis actor.

## 5. Health-Beneficial Effects of Postbiotics in Livestock

Postbiotics offer numerous health benefits for livestock, such as improving growth performance, strengthening gut barrier function, and enhancing immune response. They can also contribute to better feed conversion, potentially reducing the use of antibiotics and serving as an alternative to antibiotics.

### 5.1. Postbiotics in Poultry

**Figure 3:**
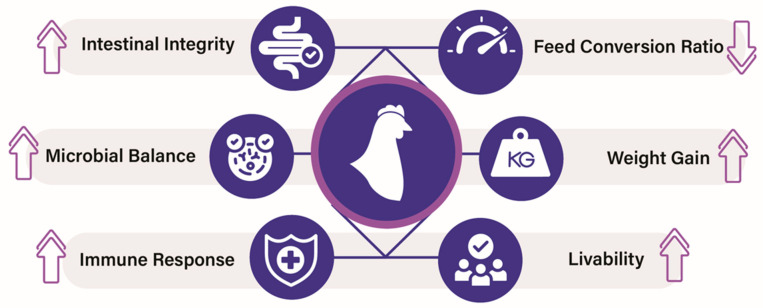
Beneficial effects of postbiotics in poultry. ↑ arrow indicates increase and ↓ arrow indicates decrease.

Poultry industries have many challenges in increasing productivity and maintaining the birds’ health. Recently, postbiotics have shown an effectiveness in poultry production, which positively impacted poultry health and performance (Figure 3). In a study, an SCFP feed additive increased turkey performance. It has been noted that SCFP alone or in combination with saponins increased the body weight of birds. Postbiotics also improved the feed conversion ratio (FCR) compared with the control group [72]. Postbiotics also prevent *Salmonella pullorum* pathogen-induced mortality in chicks. Briefly, postbiotics from *Bifidobacterium bifidum* improved growth performance and reduced mortality from 66.66% to 8.33% of chickens infected with *S. pullorum*. It also restored intestinal barrier function by upregulating the expression of tight junction proteins, including zonula occludens-1 (ZO-1), Occludin, and Claudin-1. Additionally, the cecal microbiota diversity was also improved by this postbiotic, with a decrease in the abundance of harmful bacteria (e.g., Escherichia and Shigella) and an enrichment of beneficial bacteria (e.g., *Lactobacillus* and *Ruminococcus*). Thus, *Bifidobacterium bifidum* postbiotics provide significant protection against *S. pullorum* infection by modulating pyroptosis, protecting the intestinal barrier, and restoring microbial balance [54].

Postbiotics (0.2%) from *L. acidophilus* have also shown a remarkable efficacy regarding an increase in body weight, better FCR, and stimulating an immune response in broiler chicks. Postbiotic supplementation resulted in an increase in villus height, width, and crypt depth, along with high jejunal antioxidant values. In addition, postbiotics also reduced harmful bacteria and increased *Lactobacillus* counts in broilers [73]. Humam et al. also reported that feeding with different strains (RI11, RS5, UL4) of *L. plantarum* postbiotics increased the growth performance of birds. The addition of RI11 postbiotics to birds’ diets showed an increase in final body weight, total weight gain, average daily gain, and feed conversion ratio. Postbiotic supplementation also improved villi height significantly in the duodenum, jejunum, and ileum. The postbiotic-supplemented birds had higher total bacteria and *Lactobacillus* counts and *Bifidobacterium* populations, along with lower *Salmonella*, Enterobacteriaceae, and *E. coli* counts. Besides these, plasma IgM and IgG levels were found to be significantly higher in the birds receiving postbiotics, leading to a lower mortality [70]. These findings suggest that postbiotics could be used as a potential alternative to antibiotic growth promoters and anti-stress treatment in the poultry industry.

The remarkable antimicrobial activity of *Lactiplantibacillus plantarum* postbiotics was demonstrated through its beneficial effects on *Salmonella*-challenged broilers. The oral supplementation of postbiotics (0.8%) caused an attenuation of *Salmonella*-induced intestinal mucosal damage. Postbiotics also decreased the intestinal injury score, increased villus length and villus/crypt depth, regulated the expression of intestinal injury-related genes (Villin, matrix metallopeptidase 3, intestinal fatty acid-binding protein), and enhanced tight junctions (ZO-1 and Claudin-1). Additionally, *L. plantarum* postbiotics improved the inflammatory response by decreasing IL-1β, IL-6, TNF-α, toll-like receptor 4 (TLR4), myeloid differentiation factor 88 (MyD88), and NF-κB and upregulating IL-10. Besides these, *L. plantarum* postbiotics regulate the gut microbiota by enhancing the percentage of *Lactobacillus* [74]. The Lactobacilli postbiotic, along with a probiotic, also alleviated impaired performances and the health of broilers fed with a challenged diet. Along with increasing body weight, postbiotics increased plasma magnesium in broilers [75].

Postbiotics are found to be beneficial in *Clostridium perfringens* (*C. perfringens*)-induced necrotic enteritis in broilers. The supplementation of *Lactobacillus* species postbiotics (dry and aqueous) markedly decreased the severity of necrotic enteritis (NE) signs and the mortality caused by *C. perfringens*. This postbiotic also improved the feed conversion ratio and increased hemagglutination inhibition antibody titers for the Newcastle disease virus vaccine, indicating its high immune response to pathogens [76]. In concurrence with this, the positive effects of in ovo and post-hatch applications of a SCFP were also observed. Postbiotic treatments in ovo and in drinking water improved the average daily gain of chicks, with a reduction in NE-induced lesion scores in the jejunum and ileum. The abundance of nutrient transporters SGLT1, GLUT2, and EAAT3 were also greater in SCFP postbiotic-treated chicks [77]. Enzymatically treated yeast probiotics have also demonstrated their ability to alleviate the deleterious effects of coccidia infections in poultry production. Moreover, dietary yeast postbiotics improved the growth-to-feed ratio in coccidia-challenged chickens. Postbiotics also increased the apparent ileal digestibility of dry matter, nitrogen, and gross energy. Additionally, yeast postbiotic supplementation increased the expression of tight junction gene occluding and serum antioxidant enzymes catalase, as well as the ileal villus height-to-crypt depth ratio and ileal goblet cell density in broiler chickens [78].

Another postbiotic derived from *Bacillus subtilis* had a positive influence on body weight gain and feed efficiency in broiler chicks. The inclusion of postbiotics in the diet led to an increase in the yield of breast and leg muscles. Moreover, dietary supplementation with postbiotics (0.015%) has effectively controlled the emission of ammonia from excreta and reduced the abundance of *Salmonella* in excreta, as well as enhancing the presence of *Lactobacillus* bacteria in the broiler’s gut [79]. Postbiotics have been used as a meat preservative due to their anti-microbial properties. Postbiotics (*Pediococcus acidilactici*, *Latilactobacillus sakei*/*Staphylococcus xylosus*) suppressed the growth of total mesophilic aerobic bacteria, including *L. monocytogenes*, in chicken drumsticks without changing the color properties of the drumstick samples, suggesting that it acts as a natural preservative to reduce food-borne pathogens and extend the shelf life of poultry meat and meat products [61]. Postbiotics from yeast have a potency to negate the effects of dietary mycotoxins in broiler birds. Yeast cell wall extract and a postbiotic yeast cell wall-based blend altered the adverse effects of mycotoxin-contaminated diets by improving performance, serum biochemistry, liver function, immune response, and the architecture of intestinal villi in broiler chicks [80]. 

The postbiotic compound produced by stabilized non-viable Lactobacilli also affects the health, growth performance, immunity, and gut status of *E. coli*-challenged broiler chickens. It has been found that the postbiotic compound ameliorated the enhancement of growth performance, the boosting of immune response, the improvement of the bursa of fabricius/body weight ratio, and the reduction in the intestinal coliform count in challenged chickens when compared with challenged non-treated chickens [76,81]. *L. helveticus*-derived postbiotics have also shown effectiveness in mitigating *Salmonella gallinarum* infections in commercial layer chicks. A prophylactic treatment with a postbiotic that modulates the intestinal microbiota was efficient in reducing *S. Gallinarum* in the cecum and liver of chicks [82]. It has been observed that a postbiotic cocktail from multiple sources like *Pediococcus acidilactici*, *L. reuteri*, *Enterococcus faecium*, and *L. acidophilus* has efficacy against NE caused by *C. perfringens.*

Postbiotic cocktails have been effective in lowering *C. perfringens* counts and improving lesion scores, mortality, and weight gain in the most severely challenged birds. Moreover, the postbiotic reduces pro-inflammatory responses, predominantly affects the innate immune response, and appears anti-inflammatory and immunomodulatory. Therefore, postbiotic cocktails could be a viable alternative to antibiotics to improve poultry health in the context of *C. perfringens* pathogen challenge [83]. Besides these, the SCFP was also evaluated for its effectiveness in reducing *Salmonella* *Enteritidis* colonization in young layer pullets. Feeding SCFP to the *Salmonella* *Enteritidis*-challenged birds caused a reduced prevalence of *S. Enteritidis* in the ceca of the birds [84]. Table 5 describes the effects of various postbiotics on poultry health. 

Thus, in the poultry industry, postbiotics derived from Saccharomyces cerevisiae, Bifidobacterium bifidum, *Lactobacillus* species, and Bacillus subtilis improve growth performance, body weight gain, FCR, and immune response. The upregulation of tight junction proteins upon postbiotic administration improves nutrient absorption and disease resistance by reducing pathogenic bacterial loads. Furthermore, the antimicrobial and antioxidant properties of postbiotics extend the shelf life of poultry products. Collectively, postbiotics provide an alternative to antibiotics, offering multiple benefits for bird health, performance, and food safety.

### 5.2. Postbiotics in Cattle

**Figure 4:**
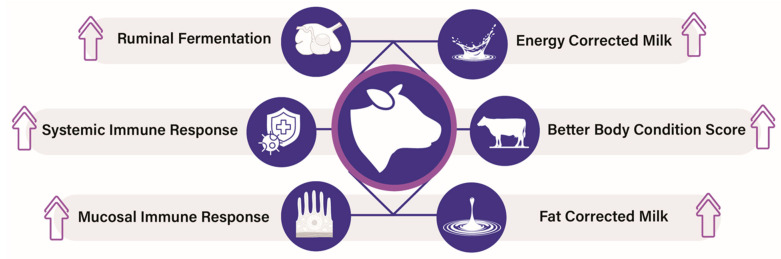
Beneficial effects of postbiotics in cattle. ↑ arrow indicates increase.

Postbiotics hold promises for improving gut health, immunity, and overall health in cattle (Figure 4). Feeding postbiotics offer a natural alternative to antibiotics and have been shown to improve milk production in cows, which potentially boosts benefits for dairy farmers. Postbiotic supplementation in late gestation and early lactation in Friesian dairy cows has been shown to increase the voluntary intake of dry matter, with a higher apparent total tract digestibility of dry matter, organic matter, and neutral detergent fiber. Postbiotics also induced an increase in the colostral immunoglobulin concentration, increased milk production with high fat and protein yields, and caused a higher persistence of the production curve throughout the lactation [86]. Besides this, dietary administration of a postbiotic from *Aspergillus oryzae* has also been shown to improve tolerance to heat stress in cattle. The supplementation of an *Aspergillus oryzae* postbiotic improved energy-use efficiency, water absorption, and intestinal permeability in heat stress-mediated increases in gut permeability, but did not reduce the heat stress-mediated rise in markers of systemic inflammation [87].

The addition of postbiotics from the SCFP to feed also improved milk production efficiency in Holstein cows naturally exposed to high temperature and humidity conditions. The cows fed with SCFP produced more milk than cows on a basal diet. SCFP-fed cows had a lower dry matter intake and greater feed efficiency, as well as energy-corrected milk. Moreover, SCFP cows showed a greater body condition score than basal diet-fed cows [88]. Thus, postbiotic feeding improves the overall health of lactating cows exposed to high temperature and humidity conditions.

A lower performance and altered immunometabolism normally occur in dairy cattle due to reduced or restricted feed intakes in their lifetime. In this aspect, postbiotics from SCFP showed a reduction in neutrophil oxidative bursts in Holstein cows during feed restriction (40% of baseline feed intake). However, feeding SCFP increased the yield of fat-corrected milk, energy-corrected milk, feed efficiency, and milk components prior to feed restriction [89]. Another study also confirmed that SCFP supplementation results in s greater energy-corrected milk yield, milk fat content, and fat-corrected milk in transition dairy cows. In addition, SCFP reduced milk urea nitrogen, lowered creatinine and cortisol concentrations, decreased plasma β-hydroxybutyrate, ceruloplasmin, haptoglobin, and IL-1β concentrations, and increased plasma phosphorus, calcium, and myeloperoxidase concentrations in dairy cows. Thus, SCFP shows multiple benefits in transition dairy cows through the modulation of immunity and liver metabolic function [90].

SCFP has also shown a positive influence on the innate immune system of cattle infected with digital dermatitis. SCFP supplementation reduced IL-1β production, particularly prior to experimental inoculations with digital dermatitis. However, during active, chronic, and focal flare-ups of digital dermatitis, SCFP supplementation resulted in a more rapid secretion of IL-1β, indicating its implications for digital dermatitis prevention and treatment [91]. The supplementing of SCFP postbiotics also modulates both systemic and mucosal immune responses in bovine respiratory syncytial virus (BRSV)- and *Pasteurella multocida*-infected calves. SCFP led to an increased quantity of IL-6 in response to toll-like receptor stimulation in the immune cells of calves. In contrast, bronchoalveolar lavage cells from SCFP-treated calves secreted fewer pro-inflammatory cytokines and less TNF-α and IL-6 in response to the same stimuli. Thus, SCFP-treated lungs are associated with a decreased expression of inflammatory responses and increased expression of plasminogen and genes involved in glutathione metabolism, supporting effective lung repair [92]. Besides this, liquid SCFP has shown the potential to prime the rumen environment and improve the subsequent ruminal fermentation and performance of Holstein steer cows. Liquid SCFP did not affect nutrient digestibility, but enhanced ruminal fermentation by increasing the total volatile fatty acid production by rumen microbes. In addition, the supplementation of SCFP enriched several plasma metabolic pathways related to energy and nitrogen metabolism, such as the citric acid cycle, pyrimidine metabolism, and glycolysis/gluconeogenesis pathways [93].

The edible coating of food materials is encouraged in food industries due to the desire for a longer shelf life and enhanced safety. In this consideration, Abbasi et al. [94] have created an edible coating using *Malva neglecta* seed polysaccharide mucilage containing *L. brevis* TD4-derived postbiotics for the preservation of beef slices. They demonstrated that the coating prevented the proliferation of microbial agents (total viable count, psychotropic count, *Staphylococcus aureus*, *E. coli*, total coliform bacteria count, and fungi) in the postbiotic-added beef slices and inhibited the oxidation of lipids in beef samples. In addition, this postbiotic-containing coating sustained the pH value, hardness, moisture, and content, maintained the meat color, prevented the quality deterioration of beef samples, and prolonged the shelf life of the meat [94]. Another study also showed that postbiotics from *Lacticaseibacillus paracasei* ZX1231 combined with a bacterial nanocellulose films significantly inhibited the fungi growth and prolonged the shelf life of beef, bread, cheese, and soy sauce [95]. It is clear that postbiotics promote cattle’s health and growth (Table 6).

Thus, postbiotics have properties in food preservation and food packaging. Altogether, postbiotics demonstrate potential in enhancing cattle health and dairy and beef production, and postbiotic-derived edible coating and packaging effectively inhibit microbial growth, lipid oxidation, and the deterioration of meat products.

### 5.3. Postbiotics in Pigs

**Figure 5:**
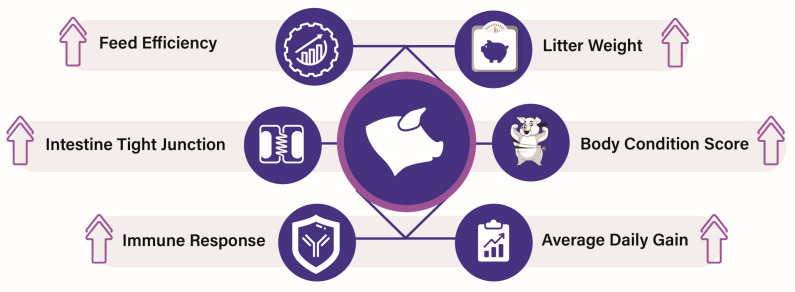
Beneficial effects of postbiotics in pigs. ↑ arrow indicates increase.

Postbiotics have been effective in enhancing gut health, boosting immunity, and improving overall performance in pigs, while offering a promising alternative to antibiotics for improving pig health and growth (Figure 5). In one study, yeast postbiotics added in feed increased the growth of pigs of all ages. Feeding SCFP with a basal diet increased the sow body score, offspring body weight at weaning, their average daily gain, feed efficiency, and average daily feed intake compared to control [96]. Yeast postbiotics also affect immune responses and oxidative stress in the jejunal mucosa of pigs. The supplementation of SCFP reduced diarrhea by providing protection to the villi in the jejunum and promoting crypt cell proliferation, as well as reducing the fecal score in newly weaned pigs. SCFP also increased the immune response by increasing the gene expression of interferon-γ (IFN-γ) in the jejunum of nursery pigs [97]. Furthermore, Hung et al. have found that SCFP supplementation has a promising effect on diarrhea incidence, small intestinal morphology, and the expression of tight junction genes Claudin-1 and Occludin in piglets. Thus, SCFP serves as a dietary supplement to enhance gastrointestinal health in piglets by strengthening the intestinal epithelial barrier and reducing pathogen translocation [98]. 

A supplementation with yeast-derived postbiotics affects sows’ performance during late gestation and lactation and their offspring’s performance. SCFP has been shown to increase the deposition of backfat in sows during late gestation and an increased average weaning weight of piglets. In addition, SCFP decreased the piglet mortality and diarrhea index. SCFP supplementation also increased the IgA content in the milk, as well as IgG and IgM content in piglet serum. The content of the total antioxidant capacity and the content of transforming growth factor (TGF)-β in saw placenta were also increased by SCFP supplementation [99].

Enterotoxigenic *E. coli* (ETEC) infection is a common causative factor for diarrhea and other intestinal disorders in humans and animals, particularly in swine. However, SCFP can alleviate the harmful effects of *E. coli*. The supplementation of SCFP ameliorated ETEC-induced diarrhea, damages in intestinal permeability and morphology, and the down-regulation of tight junctions (Claudin1, ZO-1, and Occludin). In addition, SCFP decreased oxidative stress by increasing catalase, SOD, and TAC enzymes, as well as by decreasing MDA in serum and in jejunal mucosa at similar degrees. Moreover, SCFP alleviated ETEC-induced inflammation by decreasing IL-6, TNF-α, and INF-γ and increasing IL-4 and IL-10 in serum or jejunal mucosa. SCFP also enhanced immunity by increasing serum IgG and mucosal sIgA [41]. In support to these findings, another study showed that SCFP mitigates the effects of ETEC infection and reduces the incidence of diarrhea by enhancing the microbiota and mucosal immune response in the jejunum of newly weaned pigs challenged with F18^+^*E. coli*. The supplementation of SCFP improved the fecal score compared to F18^+^*E. coli*-infected piglets. Moreover, SCFP reduced the protein carbonyl and the expression of TLR4 and increased the gene expression of the mammalian target of rapamycin, indicating its positive impact on animals’ growth and performance [100].

Besides SCFP, postbiotics from *L. acidophilus* fermentation products (LAFP) also showed efficacy in young pigs challenged with an F4 ETEC strain. A supplementation with LAFP and/or SCFP resulted in the modification of the fecal microbiome in F4-ETEC-challenged pigs, which is associated with increased liveweight, growth performance, and health status in young pigs [101]. Furthermore, Duarte et al. confirmed the efficacy of dietary *Lactobacillus* postbiotics against F18^+^*E. coli* challenge in piglets. The supplementation of *Lactobacillus* postbiotics enhanced the immunocompetence of nursery pigs by increasing the expression of IFN-γ and by reducing TLR4, NOD1, and CD14 [102]. Another postbiotic from *Pichia kudriavzevii* has also been found effective against diarrhea in piglets. It was proven that a 0.5% *Pichia kudriavzevii* FZ12 postbiotic supplementation in the diet reduced diarrhea incidence, promoted growth performance, improved gut health, and enriched beneficial bacteria, particularly *Lactobacillus* spp., in the intestines of weaned piglets, indicating that the dietary modulation through the inclusion of postbiotics may alleviate weaning-induced diarrhea [103]. 

Postbiotics from *L. fermentate* were reported to prevent post-weaning diarrhea caused by F18^+^*E. coli* in nursery pigs. The supplementation of postbiotics improved the growth performance, while enhancing the intestinal health and increasing the diversity and abundance of beneficial microbiota, in pigs challenged with F18^+^*E. coli* [104]. Dietary supplementation with *L. reuteri* postbiotics reduced the mortality rate of piglets and increased the levels of total SOD in the serum and propionic acid and butyric acid in the feces, and decreased the content of MDA in the serum. *L. reuteri* postbiotics also increased the relative abundance of Firmicutes and monoglobus and decreased the relative abundance of Bacteroides in *L. reuteri* postbiotic-treated piglets [105]. Additionally, the feeding of spray-dried powders of *L. reuteri* G7 postbiotics has been shown to ameliorate the feed/gain ratio and serum levels of D-lactate and endotoxin in weaned piglets [106].

*L. plantarum* metabolite postbiotics have beneficial effects in reducing deoxynivalenol-induced intestinal toxicity. The exposure of these postbiotics to piglets’ jejunal explants restored deoxynivalenol-induced intestinal damage, like villi fusion and atrophy, multifocal apical necrosis, and cuboid or flattened enterocytes. The *L. plantarum* metabolite also recovered the reduced density of goblet cells in the villi and crypts of jejunal explants, indicating its beneficial effects in intestinal mucosa of pigs [107]. Postbiotics from yeast products with phytogenic feed additives (curcumin and silymarin) have also been shown to improve the health, performance, and redox status of weaned piglets under the dietary challenge of mycotoxins fumonisins. Phytogenic and postbiotic combinations decreased the plasma TBARS levels and protein carbonyl, while increasing the TAC. This combination also increased the average body weight gain and decreased the mortality of piglets [108].

Antimicrobial resistance (AMR) is one of the major concerns in commercial pork production. However, SCFP and LAFP have shown efficacy against AMR *E. coli*. Postbiotics reduced the shedding of extended-spectrum cephalosporin (ESC)-resistant *E. coli* in pigs, indicating its positive impacts on reducing the carriage of resistant bacteria [63]. It has also been found that postbiotics reduce the spread of tuberculosis. In a study of wild boar (wild pigs), a supplementation with postbiotics from *Lactiplantibacillus plantarum* and *Lacticaseibacillus paracasei* reduced the presence of tuberculosis-like lesions (36.87%) and lesion severity and seropositivity (35.94%) against *Mycobacterium bovis.* Interestingly, young wild boar had a 64.72% reduction in lesions and an 81.80% drop in seropositivity, suggesting the reduced transmission of pathogens [109].

In vitro data showed that postbiotics from *L. plantarum* inhibit inflammatory markers in porcine cells. Postbiotics suppressed *Salmonella typhimurium*-induced inflammation by increasing anti-inflammatory cytokines like IL-4 and IL-10, and decreased TNF-α, IL-1β, IL-6, and IL-18. Furthermore, these postbiotics inhibited NOD-like receptor protein 3 (NLRP3) inflammasome activation, indicating their potential in preventing *Salmonella* infection [110]. Thus, postbiotics in diets have beneficial effects on different ages of pigs (Table 7). 

Overall, postbiotics represent a powerful and multifunctional feed additive for pigs that supports healthier gut microbiota, improved nutrient utilization, and a strong immunity. Its integration into swine diets not only enhances health and productivity but also aligns with global efforts towards sustainable and antibiotic-free livestock production.

## 6. Health-Beneficial Effects of Postbiotics in Companion Animals

Postbiotics offer numerous health benefits for companion animals such as dogs, cats, and horses. Postbiotics are known to cause an improved gut and immune function, better skin and coat health, and a more balanced gut microbiome (Table 8).

### 6.1. Postbiotics in Dogs

Unbalanced digestive flora remarkably affect dogs’ digestion. Postbiotics can have an effective role in balancing gut flora and improving digestion. In a study, postbiotics from *L. helveticus* and yeast strains with a mixture of baobab fruit pulp and acacia gum were administered to the Simulator of the Canine Intestinal Microbial Ecosystem model, which resulted in increased saccharolytic fermentation. Postbiotic-containing products increased health-promoting bacterial metabolites such as propionate, acetate, and butyrate, as well as increased the abundances of several saccharolytic-fermenting microbes, including *Bifidobacterium*. Conversely, proteolytic bacteria like Proteobacteria were reduced with the product compared to the control, indicating a positive modulation of the microbiome in the dog intestine [111]. The in vitro addition of postbiotics in dog fecal samples was shown to increase beneficial bacterial groups producing acetic, propionic, and butyric acids, suggesting a possible positive effect on the canine gut microbiota [112].

Elderly dogs often suffer from chronic diseases, in part attributed to poor immunity, and the supplementation of postbiotics can positively influence their immune responses. It has been found that the supplementation of yeast postbiotics containing short-chain fructo-oligosaccharides resulted in an increased CD4^+^/CD8^+^ T cell (Helper/Cytotoxic T cell) ratio in healthy senior dogs during and after vaccination against Lyme disease [113]. The immunomodulating effects of postbiotics from *L. rhamnosus* and *L. reuteri* have also been observed in the PBMC of healthy dogs. The incubation of PBMCs with postbiotics resulted in increased levels of IL-12 and IFN-γ (Th1 cytokines) and IL-10 (associated with T regulatory cells), while the levels of the Th2 cytokine IL-4 remained stable. Thus, these two Lactobacilli postbiotics stimulated canine PBMCs to produce a cytokine profile typically associated with an anti-allergic response [114].

Postbiotics like SCFP also affect the fecal characteristics and immunity of dogs undergoing transport stress. The fecal dry matter percentage was affected by diet during transport stress, but SCFP supplementation stabilized it in the dogs. Transport stress-induced COX-2 and myeloperoxidase innate immune gene expression were also stabilized in dogs fed SCFP [115]. Besides SCFP, the *Lactobacillus* fermentation product (LBFP) has positive effects on fecal characteristics and microbiota, blood biomarkers, immune function, and serum oxidative stress markers in adult dogs. LBFP supplementation caused comparatively less change in fecal scores, signifying firmer feces in treated dogs than controls. LBFP also increased fecal Faecalibaculum, Bifidobacterium, and uncultured Butyricicoccaceae in dogs. Furthermore, LBFP increased serum SOD after transport. Thus, LBFP provides benefits to dogs by stabilizing stool quality, beneficially shifting fecal microbiota, and protecting against oxidative damage when subjected to stress [116].

*Bifidobacterium animalis*, both in live probiotic and heat-treated postbiotic forms, also affected gut health in dogs. The daily supplementation of postbiotics as well as probiotics to dogs resulted in higher fecal concentrations of propionate, which is good for gut health, with multiple known positive physiological effects. These probiotics and postbiotics induced potentially beneficial changes in the abundance of functional pathways involved in pathogenicity, amino acid biosynthesis, and DNA repair [117]. 

### 6.2. Postbiotics in Cats

Postbiotics are becoming increasingly recognized as a key part of feline nutrition, particularly for digestive health and immunity. Postbiotics from *Bifidobacterium animalis* have shown positive effects on the feline microbiome. The supplementation of a control diet with postbiotics (heat-killed *Bifidobacterium animalis*) increased the abundance of membrane transport, biofilm formation, and bacterial motility pathways compared to the basal diet in cats. Similar effects were observed with probiotics [118]. Postbiotics from the *S. cerevisiae* cell wall and its active metabolites have been shown to increase the apparent digestibility of crude fiber and ash without interfering with feed consumption, fecal production, and fecal characteristics in cats. These postbiotics also reduced *C. perfringens* in cat’s feces [119].

A component of postbiotics, sodium butyrate, has been shown to positively affect the health of animals. Sodium butyrate influenced inflammation, oxidative stress, immune function, and gut microbiota in adult cats. The supplementation of 0.1% sodium butyrate in a basal diet decreased the fecal level of calprotectin, a protein released by neutrophils in response to inflammation in the gastrointestinal tract. Sodium butyrate feeding also reduced the serum levels of inflammatory TNF-α, IL-1β, and diamine oxidase. In addition, sodium butyrate supplementation in the diet increased the antioxidant GPX level and decreased lipid peroxidation. Moreover, the feeding of a sodium butyrate-containing diet resulted in a richness of gut microbiota and a high abundance of beneficial *Lachnospiraceae*, *Lachnoclostridium*, *Blautia*, and *Roseburia* bacteria [46].

### 6.3. Postbiotics in Horses

Horses are unique animals because they are considered as both farm animals and companion animals, depending on their use. Horses are used in commercial and agricultural contexts, but many owners also keep them for pleasure, sport, and emotional connection, much like pets. As per the “Center for diseases control and prevention (2025)”, horses are considered under companions. In horses, postbiotics provide a range of benefits, including improved gut health, immune function, and stress management. As nutritional, environmental, and prolonged transportation stressors disturb the gut microbiome of horses, postbiotics provide benefits for horses and improve their health and performance. SCFP has been shown to stabilize microbial profiles in horses after stress challenge. The supplementation of SCFP in upper respiratory tract-inflamed horses stabilized alpha diversity across all time points in their feces, whereas basal diet-fed horses had fluctuations [120]. An additional study also supported these findings that a SCFP-supplemented diet minimizes the increase in alpha diversity of fecal microbiota in horses vaccinated with equine influenza compared to the basal diet-fed horses [121]. This indicates that SCFP feeding alters alpha diversity and the vaccination-induced spectrum of released mediators that affect gut microbiota. 

A diet supplemented with an SCFP has an impact on the immediate response to a parenteral vaccination against influenza and tetanus in the early life of horses. An SCFP-containing diet resulted in increased numbers of the major leukocyte fractions (granulocytes, lymphocytes, monocytes, CD4^+^ T cells, CD8^+^ T cells, CD21^+^ B cells, and MHC-II^+^/CD21^−^ cells) in foals 24 h after vaccination, which remained unchanged in placebo-supplemented foals [122]. Moreover, the study suggested that the horses fed with SCFP-containing feed displayed a modulated early immune response after influenza vaccination. The supplementation of diet with SCFP displayed a significant increase in reticulocyte percentages 24 h after vaccination in horses. However, in both the SCFPO and placebo groups, the total leukocyte counts and numbers of CD4^+^ T cells significantly increased [123]. Collectively, these studies suggest that early life supplementation with an SCFP may affect the early immune response to vaccination.

**Table 8:** Various postbiotics and their effect on companion animals.

Type of Postbiotics	Dose and Route	Effect	Reference
***Dogs***			
Yeast-containing postbiotics	1.1% in feed	Increased the CD4^+^:CD8^+^ T cell ratio and decreased total serum IgA concentrations	[113]
Postbiotics from *Lactobacillus rhamnosus* and *Lactobacillus reuteri*	1 × 10^6^ tyndallized bacteria in lymphocyte-rich white blood cells from healthy dogs	Modulated the immune response and prevented of relapses of allergic diseases	[114]
SCFP postbiotics	0.13% in diet	Improved innate immune cell activation during transport stress	[115]
*Lactobacillus* fermentation product	4 mg/kg body weight	Stabilized stool quality and microbiota homeostasis and reduced oxidative damage in stress	[116]
***Cats***			
SCFP postbiotics	0.3% or 0.6%	Elevated digestibility of crude fiber and ash without interfering with feed consumption, fecal production, and fecal characteristics	[119]
Sodium butyrate postbiotic	0.1% in diet	Decreased inflammatory TNF-α and IL-1β, increased antioxidant enzymes, and improved gut microbiota	[46]
***Horses***			
SCFP postbiotics	21 g/d in diet	Stabilized microbial profiles after stress challenge	[120]
SCFP postbiotics	10 gm dry powder in diet for 28 days	Improved the early immune response to an initial vaccination	[122]

DMI—dry matter intake; IL—interleukin; SCFP—Saccharomyces cerevisiae fermentation products; TNF—tumor necrosis factor.

## 7. Conclusions

Mounting evidence suggests that postbiotics are able to confer benefits to animal health and performance. Components of postbiotics such as cell wall fractions, nuclear fractions, enzymes, short-chain fatty acids, defensins, bacteriocins, and other metabolites benefit the physiological functions of the animals. Although postbiotics are safe, contamination may cause toxicity in animal feed. For example, mycotoxin from black mold and cyanotoxins from cyanobacteria (often called blue-green algae) can cause a range of health issues, from skin irritation to severe organ damage [124]. Some of the fungi and protozoa also produce ergosterol (ergosta-5,7,22-trien-3β-ol), a sterol serving many of the same functions that cholesterol serves in animal cells. However, at high doses, irradiated sterol has shown toxicity [124]. In addition, the biological potency, safety, and stability of postbiotics present unique advantages over traditional probiotics. Although postbiotics from single microorganisms have shown a sufficient potency on animals’ health, the combined administration of either postbiotics obtained from different microorganisms or postbiotics with probiotics is more effective than individual supplementation, especially in multi-target health applications. Thus, postbiotics alone or in combination with other agents like probiotics or phytochemicals represent a promising edge in postbiotics-based health support and intervention. However, there are still some missing links between the laboratory findings and robust clinical evidence with commercial products. Therefore, multidisciplinary collaboration is required among microbiologists, clinicians, nutritionists, and regulatory bodies to unlock the full potential of postbiotics.

## Figures and Tables

**Table 5:** Effect of various postbiotics with dose and route on poultry.

Type of Postbiotics	Dose and Route	Effect	Reference
SCFP feed additive	1.25 kg/mT with basal diet	Improves body weight and feed conversion ratios	[72]
*Bifidobacterium bifidum* postbiotics	Inactivated 1 × 10^9^ CFU/mL daily via oral gavage	Reduces *S. pullorum* pathogen-induced mortality from 66 to 8%; upregulates tight junction proteins in the intestine	[54]
Postbiotics derived from *Lactobacillus acidophilus*	0.2%, 0.4%, and 0.6% with basal diet	Increases body weight, FCR, immune response, villus height and width, and crypt depth	[73]
*L. plantarum* postbiotics	0.3% postbiotics	Increases plasma IgM level, hepatic GHR, and IGF-1 mRNA, less mortality; works as a growth promoter and anti-stress treatment	[85]
*Lactiplantibacillus plantarum* postbiotic	0.8% postbiotics	Attenuates *Salmonella enterica*-induced intestinal mucosal damage; inhibits Caspase-1, IL-lβ, and IL-18 and NF-κB inflammatory pathway	[74]
*Lactobacillus acidophilus* species fermentation product	1 kg/ton-starter, 500 gm/ton-grower, and finisher feed as dry form and 4 mL/L drinking water	Decreases severity of necrotic enteritis, improves FCR, and increases hemagglutination inhibition antibody titers	[76]
SCFP postbiotic	1.6 mL product in 1 L of drinking water	Improves the average daily gain of chicks with a reduction in necrotic enteritis-induced lesion scores in the jejunum and ileum	[77]
Yeast postbiotics	1 or 2 gm/kg yeast postbiotics with diet	Reduces IL-1β; increases tight junction gene and serum catalase	[78]
Postbiotic derived from *Bacillus subtilis*	Diet containing 0.015% postbiotic	Elevates serum albumin and total protein contents, reduces the abundance of *Salmonella* and ammonia emission in excreta, and enhances *Lactobacillus* bacteria presence	[79]
Postbiotics from *Pediococcus acidilactici* and *Latilactobacillus sakei*/*Staphylococcus xylosus* (1:1 mix)	5% and 10% postbiotics in water	Decreases *L. monocytogenes* counts as well as total mesophilic aerobic bacteria counts in chicken drumsticks	[61]
Lactobacilli postbiotics	1 kg/ton-starter, 0.5 kg/ton-finisher diet, 4 mL/L in drinking water	Enhances growth performance, boosts immune response, and reduces total intestinal coliform count	[81]
Postbiotics from *E. acidilactici*, *L. reuteri*, *E. faecium*, and *L. acidophilus*	1 ounce/gallon of postbiotics in drinking water	Improves performance; reduces mortality in *C. perfringens* pathogen-challenged birds by improving innate immune response and reducing the pro-inflammatory responses	[83]

CFU—Colony forming unit; FCR—feed conversion ratio; GHR—Growth Hormone Receptor; IGF-1—Insulin-like Growth Factor-1; IL—interleukin; mT—metric ton; SCFP—Saccharomyces cerevisiae fermentation products.

**Table 6:** Different postbiotics and their effect on cattle.

Type of Postbiotics	Dose and Route	Effect	Reference
*Aspergillus oryzae* postbiotic	3.0 g/calf/day of postbiotic in milk	Improved energy-use efficiency, water absorption, and intestinal permeability in heat stress-mediated calves	[87]
SCFP postbiotic	19 gm/day in diet	Lower DMI, greater feed efficiency, and energy-corrected milk when exposed to high temperature and humidity conditions	[88]
SCFP postbiotic	19 gm/day for 9 week	Greater DMI and higher fat, ECM, and FCM yields and feed efficiency with postbiotics; reduces neutrophil oxidative stress	[89]
SCFP postbiotic	1 gm/day in feed	Systemic and mucosal immune responses	[92]

DMI—dry matter intake; ECM—energy-corrected milk; FCM—fat-corrected milk; SCFP—Saccharomyces cerevisiae fermentation products.

**Table 7:** Effect of various postbiotics containing diet/feed on pigs.

Type of Postbiotics	Dose and Route	Effect	Reference
*Saccharomyces* yeast postbiotics	175 gm/ton in diet	Reduced diarrhea after weaning and enhance immune responses	[97]
SCFP postbiotics	2.0 kg/mT in diet	Reduced diarrhea incidence and improved intestinal integrity	[98]
Yeast-derived postbiotic	1.25 or 2 gm/kg in diet	Decreased mortality and diarrhea index and increased IgG and IgM in piglets; increased MDA, lactose, and IgA content in lactating sows	[99]
SCFP postbiotics	1.25 or 2 g/kg in diet	Enhanced antioxidative capacity; decreased systemic inflammation and diarrhea	[41]
*Lactobacillus* postbiotics from inactivated *L. fermentum* and *L. delbrueckii*	2 kg/ton feed	Enhanced the immunocompetence of nursery pigs	[102]
*Lactobacillus fermentate* postbiotics	2 kg/ton feed	Improved growth performance, enhanced intestinal health, and increased diversity and abundance of beneficial microbiota in pigs challenged with F18^+^*E. coli*	[104]
*Lactobacillus reuteri* postbiotics	500 mg/kg in diet	Reduced the mortality rate and decreased oxidative stress	[105]
*Lactobacillus plantarum* postbiotics	55 gm/Littre in water	Restored the deoxynivalenol-induced intestinal damage	[107]
*Lactobacillus acidophilus* fermentation product SCFP postbiotics	2000 ppm in diet	Reduced the shedding of ESC-resistant *E. coli*	[63]

DMI—dry matter intake; ESC—extended-spectrum cephalosporin; FCR—feed conversion ratio; IL—interleukin; MDA—Malonaldehyde; mT—metric ton; SCFP—Saccharomyces cerevisiae fermentation products.

## Data Availability

No new data were created or analyzed in this study. Data sharing is not applicable to this article.
